# MicroRNAs in Cardiac Diseases

**DOI:** 10.3390/cells8070737

**Published:** 2019-07-18

**Authors:** Robin M.W. Colpaert, Martina Calore

**Affiliations:** IMAiA-Institute for Molecular Biology and RNA Technology, Faculty of Science and Engineering, Faculty of Health, Medicine and Life Sciences, Maastricht University, 6229 ER Maastricht, The Netherlands

**Keywords:** heart, miRNA, cardiac diseases, inherited cardiomyopathies

## Abstract

Since their discovery 20 years ago, microRNAs have been related to posttranscriptional regulation of gene expression in major cardiac physiological and pathological processes. We know now that cardiac muscle phenotypes are tightly regulated by multiple noncoding RNA species to maintain cardiac homeostasis. Upon stress or various pathological conditions, this class of non-coding RNAs has been found to modulate different cardiac pathological conditions, such as contractility, arrhythmia, myocardial infarction, hypertrophy, and inherited cardiomyopathies. This review summarizes and updates microRNAs playing a role in the different processes underlying the pathogenic phenotypes of cardiac muscle and highlights their potential role as disease biomarkers and therapeutic targets.

## 1. Introduction

Heart diseases are among the leading causes of morbidity and mortality worldwide [1]. Correct embryonic development, homeostasis, contraction-excitement coupling, and stress response of the cardiac muscle is controlled by precise spatiotemporal gene regulation. Alterations in these genetic expression patterns have been related to pathological cardiac conditions, which can lead to heart failure [2,3].

**Figure 1:**
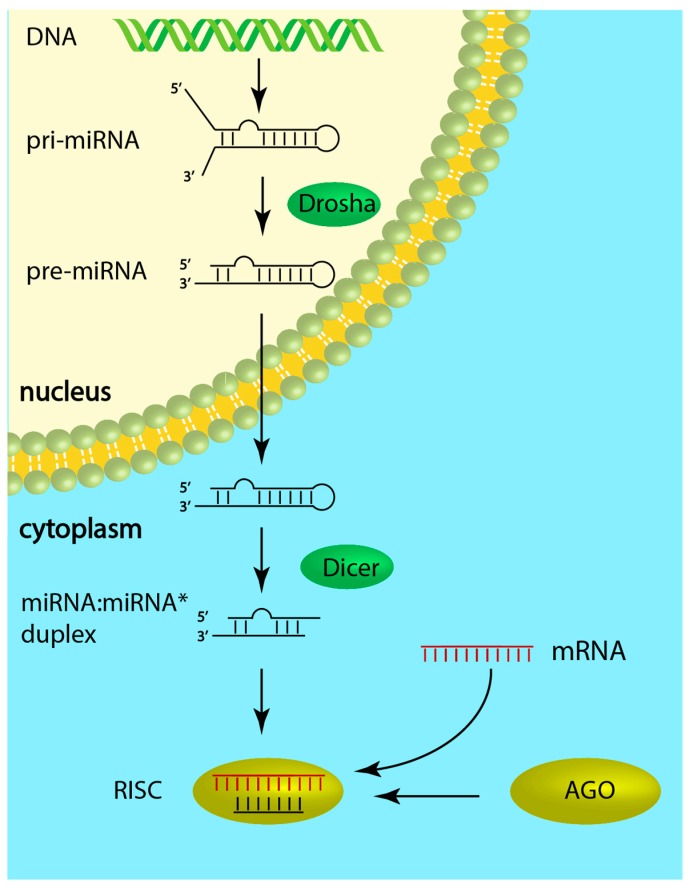
MicroRNA biogenesis. First the microRNA (miRNA) gene is transcribed to create the pri-miRNA, a single-stranded RNA hairpin with imperfect base pairing. Then, Drosha will cleave the pri-miRNA into a 70-nucleotide hairpin with a 2-nucleotide-3′ overhang, the pre-miRNA. After export to the cytoplasm, Dicer will further process the molecule and form a double-stranded miRNA:miRNA* duplex 22 nucleotides long. One strand of this duplex, called the guide strand, combines with the Argonaute (AGO) protein and the target messenger RNA into the RNA-induced silencing complex (RISC). The other strand, the passenger strand, is degraded.

In addition to the regulation by transcription factors, microRNAs (miRNAs) are also involved in differential gene expression found in the pathophysiologic cardiac condition [4,5]. MiRNAs are evolutionarily conserved noncoding RNA molecules (~22 nucleotides long, single-stranded) that regulate gene expression through imperfect base-pairing with complementary sequences in their target mRNA leading to translational repression or transcript degradation [6]. Most miRNA genes are transcribed by RNA polymerase II from intergenic, intronic or polycistronic loci as a long primary miRNA transcript (pri-miRNA), which is then cleaved by the Drosha endoribonuclease to a 70-nt-long hairpin structure with 2-nt-3′ overhangs (pre-miRNA) [7]. Pre-miRNA is subsequently exported to the cytoplasm and processed by a second endoribonuclease, Dicer, to form a 22-nucleotide-long miRNA:miRNA* duplex with imperfect complementarity. One strand of this duplex, the guide strand, then combines with the Argonaute (AGO) protein into the RNA-induced silencing complex (RISC), while the passenger strand gets degraded [8]. Whether the 5p or the 3p (i.e., originating from the 5′ or 3′ end of the pre-miRNA hairpin) RNA strand of the duplex becomes the guide strand depends partially on the thermodynamic stability at the 5′ ends of the duplex [9]. In general, the strand with lower stability preferentially combines with the AGO (Figure 1).

This leads to certain miRNAs having both their strands loaded into the RISC with equal proportion, while for others one strand will dominate [9]. The targeting of a mRNA occurs through imperfect base-pairing between the transcript and the so-called seed sequence in the miRNA, usually covering the nucleotides in positions 2–7 of the latter [10]. As a consequence, a single miRNA can regulate multiple mRNA targets involved in diverse biological processes and, vice versa, a single mRNA can be regulated by several miRNAs.

Mature miRNAs are referred to with the prefix “miR-” followed by an identifying number reflecting their order of discovery. In case of miRNAs with sequences differing in only one or two nucleotides, an additional letter or number is added to the name, for example ‘miR-208a’ versus ‘miR-208b’ or ‘miR-133a-1′ versus ‘miR133a-2′ [4,7].

MiRNAs can be grouped into families, based on the mature miRNA or on the sequence and/or structure of the pre-miRNAs [11]. Many miRNAs are also grouped together into polycistronic clusters, in which several miRNAs are produced from one primary transcript [12]. Up to 60% of human protein coding genes are estimated to be post-transcriptionally regulated by miRNAs [13] and, currently, ~2500 miRNAs have been annotated in the human genome [14]. In recent years, the role of miRNAs in the control of cardiovascular events related both to key biological functions and disorders has been increasingly investigated.

**Figure 2:**
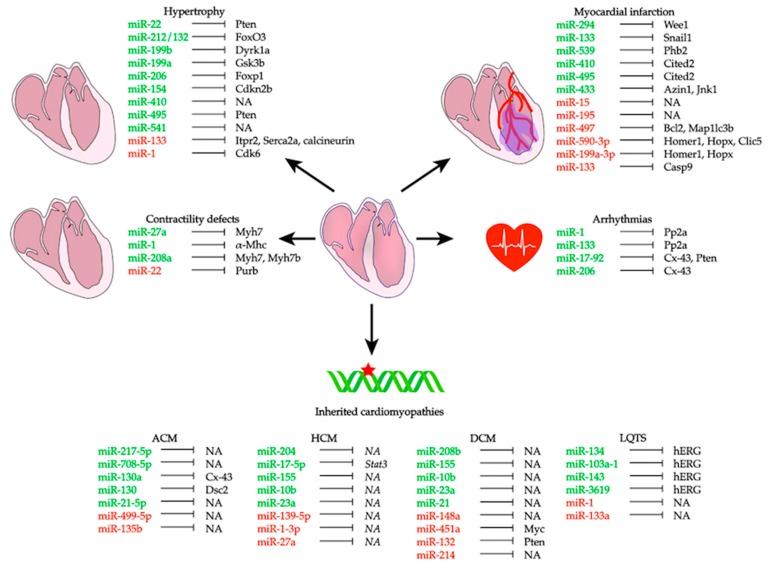
MiRNAs and the relative targets associated with cardiac diseases. Green miRNAs are upregulated, while red miRNAs are downregulated in the different diseases. *NA*, not available.

**Figure 3:**
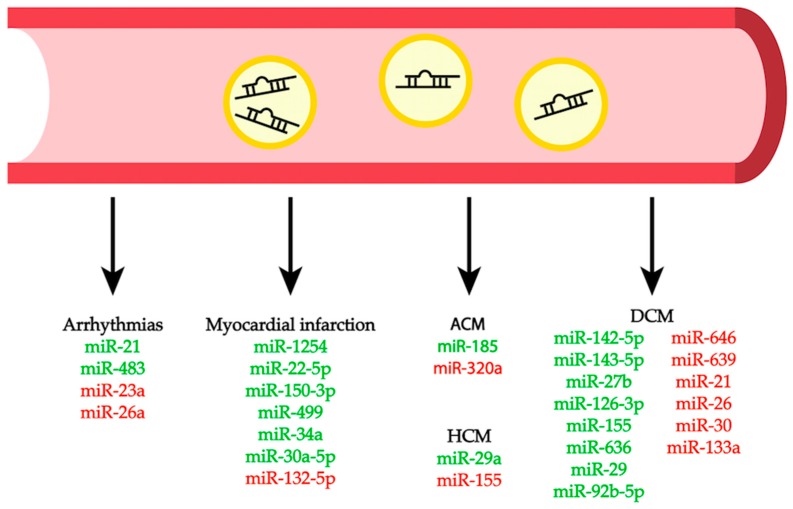
Circulating miRNAs are associated with different cardiac diseases. Green miRNAs are upregulated, while red miRNAs are downregulated in the different diseases.

This review summarizes the most recent studies highlighting the role of miRNAs in several cardiac pathogenic conditions (Table 1, Figure 2). We also provide an overview of the potential role of circulating miRNAs as disease biomarkers (Figure 3), as well as a description of the currently available approaches to use miRNAs as potential therapeutic tools for different cardiac conditions. This review limits its scope to studies of the myocardium itself; the roles of miRNAs in other types of cardiovascular diseases, like those involving the vasculature, diabetes, or aging, are not included.

## 2. Contractility Defects

Cardiac contractility consists of the fast and unidirectional development of mechanical force and motion, determined by a highly ordered organization of sarcomeric proteins. The progressive impairment of passive extension and active contraction after adverse structural remodeling (e.g., concentric hypertrophy) is an indicator of heart failure [87,88]. Cardiac contractility primarily relies on the expression of two cardiac myosin heavy chain (MHC) genes, α and β, also known as *Myh6* and *Myh7*, respectively, which are regulated in an antithetical manner [89]. Together with cardiac stress, which induces the parallel downregulation of *Myh6* and the upregulation of *Myh7*, endogenous factors regulate the expression of these genes. Thyroid hormone T3 signaling controls the expression of these two MHC genes by stimulating *Myh6* expression and inhibiting *Myh7* expression after birth [90]. In addition to the thyroid hormone signaling, some miRNAs have been related to the regulation of *Myh6* and *Myh7* genes. In vitro assays coupled to bioinformatic analysis showed that miR-27a targeted *Myh7*, but not *Myh6*, by inducing a strong upregulation of the gene upon thyroid hormone receptor β1 (*Tr*β*1*) signaling in neonatal rat ventricular cardiomyocytes and mouse embryonic stem cells [15]. In vivo studies in hypertrophic hearts in mice that underwent transverse aortic constriction (TAC) further highlighted this relationship, with the parallel upregulation of miR-27a as well as of *Myh7* and the downregulation of *Tr*β*1* [15].

Several studies also highlighted an important role of miR-208 in the regulation of MHC genes. MiR-208a is encoded by an intronic portion of *Myh6* gene and its deletion in mice was related to the decreased expression of *Myh7* gene in response to stress or hypothyroidism, after thoracic aortic banding [16]. A second study in mice further highlighted the regulatory role of miR-208a, which modulated the expression of *Myh7* and *Myh7b*, as well as of miR-208b and miR-499, their respective intronic miRNAs. In turn, miR-208b and miR-499 demonstrated a dominant role in the specification of muscle fiber identity by activating slow and repressing fast myofiber gene programs [91]. Another study showed an increase in the expression of pro-hypertrophic β-MHC during early stages of diabetes in type-2 diabetic mouse hearts following the upregulation of miR-208a [17]. The upregulation of miR-208a appeared to precede the switch from α- to β-MHC isoforms and the development of systolic and diastolic dysfunction. Inhibition of this miRNA prevented the activation of β-MHC and the subsequent hypertrophic response. A similar upregulation of miR-208a was found in tissue of type-2 human diabetic hearts [17]. Taken together, these studies highlight the double role of MHC genes, which, in addition to producing a major cardiac contractile protein, regulate cardiac growth and gene expression in response to stress and hormonal signaling through miRNAs encoded in their introns.

Other studies reported other miRNAs involved in the regulation of cardiac contractility, with mechanisms not related to MHC. Mice under pressure overload conditions display a reduction of miR-22, which in turn has been related to the alteration of the intracellular calcium homeostasis due to a reduced sarcoplasmic reticulum (SR) Ca^2+^ load [18]. In these conditions there was a decrease in the expression of sarcoplasmic reticulum Ca^2+^ ATPase activity (*Serca2a*), a Ca^2+^ reuptake pump critical for cardiac contractility and whose reduced expression is a marker for heart failure, as well as of several genes encoding for proteins in the vicinity of the cardiac Z disk/titin cytoskeleton: *Calsarcin-1, Casq2, Ldb3, Melusin* and *Titin*. Furthermore, in miR-22^-/-^ mice the transcription factor *Purb* is upregulated; this in turn negatively regulates *Srf*, itself a transcription factor that modulates *Serca2a* expression. This suggests that miR-22 is involved in the impaired contractility through *Serca2a* regulation [18]. Additionally, a transgenic mouse model for cardiac-specific overexpression of miR-1 showed age-dependent decrease of heart function associated with myofibril fragmentation and shorter sarcomeres [19]. Downregulation of genes involved in sarcomeric assembly, such as myosin light chain kinase (*Mylk3*) and cardiac calmodulin (*Calm1/Calm2*), was observed. This led to decreased phosphorylation of myosin light chain 2v (MLC2v), cardiac myosin binding protein-C (cMyBP-C) and calmodulin-activated protein kinase II (CaMKII). In the suggested mechanism, miR-1 overexpression led to the repression of *Mylk3* and *Calm1/Calm2* genes, which in turn attenuated the phosphorylation of MLC2v, CaMKII, and cMyBP-C, with subsequent sarcomeric disassembly, adverse structural remodeling, and impaired heart function [19].

## 3. Arrhythmias

Arrhythmias are abnormal deviations from the normal heart rate and/or rhythm and are generally caused by abnormal conduction or repolarization, or by a combination of both [92]. In diseased hearts, regional changes in electrophysiology can lead to non-uniform anisotropy of impulse propagation, and anisotropic re-entry lies at the basis of arrhythmic development [93]. Cardiac arrhythmias may arise from alterations in intracellular Ca^2+^ cycling in the SR. Normally, due to depolarization, Ca^2+^ enters the cardiomyocytes through voltage-dependent Ca^2+^ channels during the plateau phase of the action potential, which then triggers a release of more Ca^2+^ from the SR. This process is controlled through the phosphorylation of phospholamban (SR Ca^2+^-ATPase inhibitor), the ryanodyne receptor (RyR2) (the SR Ca^2+^ release channel) and the L-type Ca^2+^ channel, a process that has been shown to be regulated by miRNAs [94,95]. Overexpression of miR-1 in rat ventricular cardiomyocytes resulted in abnormal Ca^2+^ cycling and showed increased phosphorylation of RyR2, while decreasing the expression of a subunit of protein phosphatase 2A (PP2A), a phosphatase which can influence cardiac contractility through phosphorylation of proteins necessary for Ca^2+^ release [20]. Similarly, in cardiomyocytes of hearts isolated from canines with chronic heart failure that showed increased left ventricular (LV) dimensions and reduced LV contractility, the expression of both miR-1 and miR-133 was found to be increased [21]. Again, decreased PP2A, increased RyR2 phosphorylation and abnormal Ca^2+^ cycling were responsible for the phenotypic changes [21].

Arrhythmias can also be caused by abnormalities in the function of gap junctions, intercellular structures in the intercalated discs which provide a low-resistance pathway for direct cell-to-cell passage of the electrical stimulus [96]. Connexin-43 (Cx-43), encoded by the gap junction alpha-1 (*GJA1*) gene, is the most important component of the gap junction in the heart. The miRNA cluster miR-17-92 targets this gene, as well as the phosphatase and tensin homolog (*Pten*) gene, a lipid phosphatase that plays a role in cardiomyocyte size and cardiac contractility [22]. Conditional overexpression of the miRNA cluster in cardiac and smooth muscle tissues in a homozygous transgenic mouse model led to downregulation of these two genes resulting in spontaneous arrhythmias and a dramatic decrease in survival rate [22]. The authors hypothesized that the direct suppression of *Gja1* by miR-17-92 in the heart may aberrantly regulate the electric impulse propagation with subsequent lethal arrhythmias. Cx-43 was also found to be a direct target of miR-206 [23]. The overexpression of this miRNA in HL-1 cells and in adult mouse heart caused suppression of Cx-43, abnormal heart rate and PR interval, and reduced life span in the animals.

## 4. Myocardial Infarction

Myocardial infarction (MI) refers to a decrease in blood flow to the heart, resulting in damage to cardiomyocytes due to lack of oxygen and it is often caused by the rupture of atherosclerotic plaques [97,98]. Acute MI is characterized by ischemic injury and cardiomyocyte apoptosis, followed by structural alterations, such as increase of extracellular matrix protein, fibrosis, and hypertrophy of cardiac myocytes, which leads to cardiac dysfunction and eventually causes heart failure [99]. Although the heart was long considered to be a non-mitotic organ, studies performed on cardiac tissue samples from patients who died 4 to 12 days after MI showed that 4% of myocyte nuclei express the proliferation marker Ki-67 in the regions adjacent to the infarct [100]. Interestingly, cardiomyocyte proliferation was shown to be involved in cardiac regeneration and both processes have been related to miRNA regulation. Several studies detected miRNAs as key regulators of cardiomyocyte proliferation and of improvement of cardiac function after MI. While miR-15, miR-195, and miR-497 were reported to negatively regulate these processes [24,25,26], miR-590-3p, miR-199a-3p and miR-294 are considered promoters of cell cycle re-entry in both neonatal and adult rat cardiomyocytes as well as in in vivo studies on MI adult mouse models [27,28].

A miRNA frequently associated with MI is miR-133. In a study on bone marrow-derived mesenchymal stem cells (MSCs), Chen et al. revealed the protective role on miR-133 against apoptosis under hypoxia [29]. Transplantation of miR-133-overexpressing MSCs in rat infarcted hearts resulted in lower inflammatory level and smaller infarct size, as well as in improved cardiac function, due to the repression of the expression of the fibrinogenesis-promoting gene 1 (*Snail1*) in cardiomyocytes [29]. A previous study, focused on the role of the β-blocker carvedilol in MI, showed that the upregulation of miR-133 by this drug ameliorates the impaired cardiac function and reduced apoptosis in the infarcted heart in rats and in the presence of oxidative stress [30]. The cytoprotective role of carvedilol was abolished by knocking down miR-133 by its antisense inhibitor, while it was mimicked by the overexpression of the miRNA, which reduced apoptosis by targeting Caspase-9, one of the first molecules activated in the apoptosis [101]. Very recently, the role of miR-133 in MI was further studied in cardiomyocytes or in hearts of mice treated with the anthraquinone aloe-emodin (AE) [31]. In this context, H_2_O_2_-mediated downregulation of miR-133 was inhibited by AE, which also prevented the increase of Caspase-3 activity. Accordingly, transfection with miR-133 inhibitor abolished the anti-apoptotic effects of AE. These results suggest the protective role of miR-133 in MI and corroborate its function in the prevention of apoptosis.

A group of miRNAs implicated in the cardiac response to MI maps in the so-called *Dlk1*-*Dio3* noncoding RNA (ncRNA) locus [102,103]. This evolutionary conserved genomic region spans between the Delta-like homolog 1 (*Dlk1*) and type III iodothyronine deiodinase (*Dio3*) protein coding genes and hosts a large cluster of ncRNAs, including more than 50 miRNAs both in mice and in humans, as well as several long noncoding RNAs (lncRNAs) and small nucleolar RNAs [103]. Interestingly, immediately following the *Dlk1* gene there are two intergenic differentially methylated DNA regions [104]. This epigenetically imprinted region regulates gene expression of the locus, with the methylated paternal allele expressing the protein-coding genes and the unmethylated maternal allele expressing the ncRNAs. One early study characterized the miRNA expression profile of a MI mouse model and found that more than a quarter of all differentially expressed miRNAs belonged to the *Dlk1*-*Dio3* region [102]. All of these miRNAs were upregulated. Several reports corroborated these findings. Two independent studies showed that miR-539 is upregulated in response to anoxic conditions in vitro [32] and MI in vivo [33]. In the former, Wang et al. showed that, in MI mice, the cardiac apoptosis-related lncRNA (*Carl*) can suppress apoptosis and mitochondrial fission in anoxic cardiomyocytes by acting as a ‘sponge’ for miR-539, which in turn targets prohibitin-2 (*Phb2*), a gene important for mitochondrial function [32]. In the latter study, miR-539 was shown to target *O*-GlcNAcase (*Oga*), a protein-targeting deglycosylase that was downregulated in a MI mouse model [33]. Increased global levels of protein *O*-GlcNAcylation has been shown to improve cardiac response after acute stress such as ischemia-reperfusion, MI and oxidative stress. [105]. Two other miRNAs belonging to the *Dlk1*-*Dio3* region are miR-410 and miR-495. These were shown to be able to promote proliferation of cardiomyocytes in vitro through targeting Cbp/P300 Interacting Transactivator With Glu/Asp Rich Carboxy-Terminal Domain 2 (*Cited2*), a transcriptional co-activator that plays a role in cardiac development and morphology [34]. A subsequent study by the same group showed that these two miRNAs are upregulated in mouse models for MI, cardiac hypertrophy and muscular dystrophy [35]. Finally, another study found increased expression of miR-433 in three models of cardiac injury featuring fibrosis [36]. Treatment of miR-433 antagomir before inducing MI in the animals led to better preservation of cardiac function and reduced fibrosis. MiR-433 downregulated antizyme inhibitor 1 (*Azin1*) and c-Jun N-terminal kinases 1 (*Jnk1*)*,* leading to activation of the transforming growth factor-β (TGF-β) pathway and the mitogen-activated protein kinase 1 (*Mapk1*).

## 5. Hypertrophy

Cardiomyocyte hypertrophy (CH) indicates an increase in the size of cardiomyocytes without an increase in the amount of cells [106]. While heart muscle hypertrophy initially represents an adaptive response necessary for the maintenance of the cardiac output, prolonged hypertrophic growth is associated with adverse molecular and histological consequences. This often results in heart failure through cardiomyocyte degeneration and death [107,108]. Hypertrophy can either be physiologic, such as when it develops during exercise, or pathologic, when it leads to heart failure and other cardiovascular diseases. CH, hence, is a highly complex remodeling process, also regulated by miRNAs. Several reports support the pro-hypertrophic role of miR-22. Its cardiac-specific deletion in mouse was sufficient to blunt hypertrophy and cardiac remodeling in the presence of stressors, such as isoproterenol and activated calcineurin transgene [37]. Additionally, miR-22 overexpression in neonatal rat cardiomyocytes increased cell size and induced hypertrophic markers, such as the natriuretic peptide A (*Nppa*) gene, while knockdown of miR-22 attenuated the hypertrophy induced by phenylephrine, isoproterenol or angiotensin II (Ang-II) [45]. The Authors showed that miR-22 might influence CH by *Pten* inhibition, possibly involving phosphatidylinositol-3-kinase (PI3K)-protein kinase B (AKT) [45]. This data agrees with the findings observed both in neonatal rat cardiomyocytes as well as in a mouse model for CH obtained through agomir-22 treatment [39]. The upregulation of miR-22 not only induced CH, but also increased the production of the hypertrophic markers atrial natriuretic peptide (ANP), brain natriuretic peptide (BNP), and β-MHC, and reduced PTEN protein levels.

The miR-212/132 family was found upregulated in cardiomyocytes following different hypertrophic stimuli such as Ang-II, phenylephrine or insulin-like growth factor and in the cardiac tissue of TAC mice [40]. While miR-212/312 overexpression turned out to be sufficient to induce CH in transgenic animals, miR-212/132 null mice presented a smaller heart-to-body-weight ratio than wild type (WT) animals and were protected against CH induced by TAC operation [40]. Both miRNAs appeared to target and negatively regulate the expression of the anti-hypertrophic forkhead boxO (*FoxO3)* transcription factor, with the subsequent hyperactivation of the pro-hypertrophic calcineurin/nuclear factor of activated T cell (NFAT) signaling pathway [40]. The same pathway correlated also with miR-199b, which was found upregulated not only in hearts from calcineurin transgenic mice, an animal model for heart failure, but also in TAC mice, and in biopsies from human patients who suffered from heart failure [41]. MiR-199b resulted to be overexpressed by the calcineurin/NFAT pathway in vivo and to target the dual specificity tyrosine phosphorylation regulated kinase 1A *(Dyrk1a)* gene, involved in the phosphorylation of NFAT factors in pathogenic feed forward mechanism. Accordingly, in vivo treatment with a chemically modified antisense oligonucleotide specific for miR-199b led to regression of hypertrophy, restoration of *Dyrk1a* expression levels, and normalized NFAT activity [41].

MiR-199a is also a pro-hypertropic miRNA and its cardiomyocyte-specific overexpression in mice induced CH and inhibition of autophagy [42]. In particular, miR-199a targeted the pro-autophagic and anti-hypertrophic factor glycogen synthase kinase 3β, known to suppress the mammalian target of rapamycin (mTOR), one of the major negative autophagic regulators. Moreover, the hypertrophic induction by miR-199a was attenuated by overexpressing the autophagy related gene 5 in cardiomyocytes, while treatment with rapamycin restored cardiac autophagy and decreased hypertrophy in miR-199a transgenic mice [42].

Another well-known cardiac miRNA is miR-206, which, if overexpressed, resulted in CH in both in vitro and in vivo [43]. Indeed, its suppression exacerbated ischemia/reperfusion injury and hindered CH. Mechanistically, miR-206 is positively regulated by Yes-associated protein (YAP), a key molecule of the Hippo pathway, which induces cardiomyocytes apoptosis and hypertrophy, and targets the forkhead box P1 (FoxP1), known to negatively regulate cardiac hypertrophy through inhibition of Nfat3 [109]. As with MI, several miRNAs from the *Dlk1*-*Dio3* locus play a role also in CH. An example is given by miR-154, which was upregulated in a TAC mouse model [44]. Injection of an antimir against miR-154 reduced adverse cardiac remodeling and fibrosis after TAC. It also led to the reduced expression of cyclin-dependent kinase inhibitor 2B (*Cdkn2b*), a cell cycle inhibitor. The Authors speculated that regulation of *Cdkn2b* by miR-154 might contribute to the anti-fibrotic phenotype, although they did not show it was a direct target of the miRNA [44]. Also, miR-410 and miR-495 were upregulated in an Ang-II-induced mouse model for CH as well as in neonatal rat cardiomyocytes treated with phenylephrine, a pro-hypertrophic compound [35]. Knockdown of these two miRNAs in neonatal rat cardiomyocytes reduced the hypertrophic response after phenylephrine treatment. A later study using a rat model of pulmonary arterial hypertension found that the upregulation of miR-495 also increased hypertrophic markers, while the reduction of this miRNA attenuated the pathogenic phenotype [45]. While *Dlk1*-*Dio3* miRNAs are usually upregulated in cardiac diseases, some are downregulated in the pathological conditions. Among them, miR-541 was downregulated in cardiomyocytes treated with Ang-II, as a model of CH [46]. Transgenic mice overexpressing miR-541 had reduced hypertrophy upon Ang-II treatment. While the target of miR-541 was not identified, the Authors found that the miRNA itself was negatively regulated by the pro-hypertrophic microphthalmia-associated transcription factor (MITF) [46].

A significant number of other miRNAs have been related to the negative regulation of CH. Among them the most known are miR-1 and miR-133. MiR-1 is known to reverse CH during the compensatory phase of heart failure. Restoration of miR-1 expression via adeno-associated virus (AAV) serotype 9 delivery in Sprague-Dawley rats subjected to ascending aortic stenosis resulted in the regression of the hypertrophy, reduction of myocardial fibrosis and apoptosis as well as inactivation of the MAPK signaling pathway [47]. In a mouse model treated with isoproterenol (used to induce heart failure), miR-1a-3p agomir (agomir-1) injection reduced CH, fibrosis and apopotosis. Also, the mitochondrial DNA-encoded proteins NADH dehydrogenase 1 and cytochrome c oxydase 1 increased after the treatment with agomir-1, suggesting novel possible therapeutic targets for the disease [48]. The potential role of miR-1 in CH was investigated also in relation with the cyclin-dependent kinases-Retinoblastoma (CDKs-Rb) pathway [49]. While miR-1 expression was decreased in hypertrophic myocardium of rats that underwent abdominal aortic constriction and in phenypephrine-treated neonatal rat cardiomyocytes, the cyclin-dependent kinase 6 (CDK6) level was increased in the same samples and was shown to be a target of miR-1. Treatment with miR-1 mimic or CDK6 siRNA reduced the hypertrophic phenotype in terms of reduction of cell size, expression of ANF and β-MHC and the phosphorylated pRb. These results suggest a role of miR-1 in the axis CDK6-Rb pathway in CH. [47,49]. MiR-133 was one of the first miRNAs described in CH: in 2007, its expression inversely correlated to CH in transverse aortic arch-constricted mice, transgenic mice with cardiac-restricted overexpression of a constitutively active mutant Akt kinase, and rats induced to exercise [50]. More recently, miR-133, which is an antagonist of inositol 1,4,5′-triphosphate receptor II calcium channel, was found downregulated in hypertrophy, leading to increased calcium signaling and thus inducing pathological remodeling [51]. In a separate study, miR-133 expression was investigated in rats subjected to hyperthyroidism [52]. Treatment with type 1 Angiotensin II receptor (AT1R) caused elevated levels of thyroid hormone, which in turn led to CH. In this context, miR-133 was downregulated and, accordingly, its targets SERCA2a and calcineurin were upregulated. These results suggest that miR-133 plays a key role in CH through different mechanisms.

## 6. MiRNAs and Inherited Cardiomyopathies

Genetically inherited cardiomyopathies represent a significant percentage of cardiovascular diseases [110]. These include disorders such as arrhythmogenic cardiomyopathy (ACM), hypertrophic cardiomyopathy (HCM), and dilated cardiomyopathy (DCM). Several miRNAs have been found altered also in these genetic diseases.

ACM is characterized by ventricular cardiomyocyte loss and subsequent replacement by fibro-fatty tissue, often leading to severe ventricular tachyarrhythmias and sudden cardiac death [111]. Most mutations causing ACM have been identified in genes coding for desmosomal proteins [111]. In a recent study, transgenic mice overexpressing the Q558* mutation in DSG2 gene, encoding for desmoglein-2, recapitulated several ACM features, such as fibro-fatty replacement, reduction in desmosomal size and number as well as the suppression of the Wnt/β-catenin signaling [53]. RNA sequencing of heart tissue revealed that miR-217-5p and miR-708-5p were the most upregulated miRNAs, while miR-499-5p was the most downregulated miRNA in the ACM model [53]. Another study reported that overexpression of miR-130a in murine myocardium induced ventricular arrhythmias and that this miRNA targets the gap junction protein connexin-43 [54]. Interestingly, in a separate study using the same model, the Authors identified the desmosomal protein desmocollin-2 as a target of miR-130a [55]. The work of Zhang et al. is the first one investigating the expression of miRNAs in human ACM samples [56]. The evaluation by S-Poly (T) Plus of the expression of 1078 miRNAs in 24 human cardiac ACM samples identified miR-21-5p and miR-135b as significantly up- and downregulated, respectively. Interestingly, in silico analyses suggested the correlation of these two miRNAs with Wnt and Hippo pathways, which have been associated with ACM pathogenesis [112,113]. Translational functional studies focused on these miRNAs are required to confirm this data and to support the role of these ncRNAs as a potential therapeutic target for this disease.

In genetic diseases, the definition of the underlying pathogenesis is often challenging, as mutations in a given gene can be associated with different phenotypic expressions. MiRNAs, and other non-coding RNAs, represent an additional layer that should be investigated in a homogenous group of patients to clarify the mechanisms associated with perturbations in a specific gene. Within this context, a study has been performed to investigate HCM, characterized by the so-called “myocardial disarray” involving the hypertrophic nondilated left ventricle, is frequently caused by mutations in genes coding for proteins that are part of the contractile components of the cardiac sarcomere or Z disk [114]. Kuster et al. found miR-204, embedded in the transient receptor potential cation channel subfamily M member 3 (*TRPM3*) gene, to be upregulated in heart samples from 6 HCM patients carrying a mutation in the myosin binding protein C (*MYBPC3*) gene, one of the most frequently mutated genes in the disease [57]. *TRPM3* encodes for a cation-selective channel involved in calcium entry and, interestingly, was also upregulated in HCM samples. Accordingly, calcium homeostasis is dysregulated in HCM [115] and this finding suggests that *TRPM3* might be involved in the disease pathogenesis caused by *MYBPC3* mutations.

MiR-139-5p was found to be one of the most downregulated miRNAs in HCM patient hearts [58]. This result was confirmed in a recent study where the expression of miR-139-5p was reduced in left ventricular tissues of HCM patients [59].

Another study searched for miRNA-transcription factor feed-forward loops that were differentially regulated in HCM patients by integrating miRNA and gene expression profiles with experimentally verified transcription factor-target gene and miRNA-target gene interactions [60]. The Authors found that the most dysregulated feed-forward loop was between miR-17-5p and the fatty acid synthase (*FASN*) and the signal transducer and activator of transcription (*STAT3*) genes. *FASN*, a palmitate-synthesizing gene, has been linked to heart failure [116], while STAT3 is a cell-survival factor which protects cells from apoptosis during oxidative stress [117]. Interestingly, increased levels of phosphorylated STAT3 were found in a double-mutation murine model of familial HCM [118], while miR-17-5p has been shown to target *STAT3* [119].

DCM is defined by an abnormally large left ventricle with poor contractility and is genetically very heterogeneous [120]. Most of the pathogenic mutations have been found in genes encoding for proteins related to the cytoskeleton, sarcomere and nuclear envelope [120,121]. Highlighting the complex regulatory roles that miRNAs can play, miR-148a was recently found to be downregulated in DCM while being upregulated in concentric hypertrophy in human cardiac biopsies [61]. These findings were corroborated in transgenic mouse models for DCM and concentric hypertrophy. Antagomir-mediated miR-148a knockdown in WT mice led to thinning of the cardiac wall, chamber dilation, increased left ventricle volume and reduced ejection fraction. On the other hand, upregulation of miR-148a through AAV-mediated delivery protected against systolic dysfunction caused by pressure overload [61].

MiR-208b was found upregulated in the myocardium of a heterozygous knock-in mouse model expressing a truncated titin, after induction of DCM by chronic exposure to angiotensin II or isoproterenol [62]. Inhibition of miR-208b with an antimir prevented DCM development in this mouse model and led to cardiac hypertrophy and only slight fibrosis, which was similar to WT mice treated with angiotensin II. Interestingly, the study also found that miR-208b was significantly upregulated in human DCM patients [62].

Another report on CD4^+^ T cells, which are known to play a role in the chronic inflammation of DCM, found that miR-451a was downregulated in the CD4^+^ T cells of DCM patients [63]. The overexpression of miR-451a in T cells inhibited their activation and proliferation, while the inhibition of this miRNA led to increased expression of activation markers. *Myc* was determined to be a target of miR-451a and was found to be upregulated in CD4^+^ T cells from patients with DCM, while knocking down *Myc* expression also suppressed the activation and proliferation of T cells [63]. Together with cardiac fibrosis, inflammation was investigated also in another study on DCM rats [64]. The downregulation of miR-132 was associated not only to elevated apoptosis and cardiac fibrosis, but also to higher levels of PTEN, Bcl-2 associated X protein, Ang II and aldosterone inflammatory markers. On the contrary, the upregulation of miR-132 caused reduction of PTEN and activated the PI3K/Akt pathway, which resulted in the repression of apoptosis, cardiac fibrosis and inflammatory response.

While they are distinct diseases, one study using left ventricular heart samples from both DCM and HCM patients revealed that miR-155, miR-10b and miR-23a were all overexpressed in both diseases compared to control samples [65]. On the other hand, miR-214 and miR-21 were respectively down- and upregulated in DCM patients specifically, while miR-1-3p and miR-27a were found to be downregulated only in HCM patients [65]. This result suggests that miRNAs are involved in very complex regulatory networks orchestrating the development of phenotypic features typical of specific different diseases.

Interestingly, also one major component of the miRNA biogenesis machinery, *Dicer*, has been linked to DCM. Targeted cardiac deletion of *Dicer* in a mouse model led to progressive DCM, heart failure and early postnatal lethality [122]. A miRNA microarray confirmed the reduction of mature miRNA levels, while levels of miRNA precursors were increased. Consistent with these results, the expression of DICER was found to be decreased in human patients with DCM or heart failure. However, after implantation of a left ventricular assist device, the expression of DICER was restored [122].

Long QT syndrome (LQTS) is a genetic channelopathy characterized by the prolongation of ventricular repolarization, susceptibility to Torsades de pointes, and risk for sudden death [123]. Several major cardiac components were found altered in LQTS. The miR-1/miR-133a cluster includes the most abundant miRNAs in the heart and is involved in the regulation of cardiac ion channels. The decrease of the miR-1/miR-133a cluster dosage in mice was sufficient to induce the LQTS phenotype, related to the abnormal impact of the β-adrenergic signaling on the depolarizing L-type calcium channel [66]. The authors were not able to detect a single miR-1 or miR-133a target gene involved in the alteration of the adrenergic signaling, but they hypothesized that multiple changes determined by the perturbed cluster can orchestrate the L-type calcium channel activity in their LQTS model [66]. The ether-a-go-go-related gene (hERG) is the major molecular component of the rapidly activating delayed rectifier K^+^ current (Ikr) and it is also modulated by miRNAs in LQTS. By coupling in silico and in vitro studies, Lian et al. correlated miR-134, miR-103a-1, miR-143, and miR-3619 overexpression with the downregulation of hERG mRNA and protein [67]. Moreover, the current activation and tail amplitude of hERG channel was reduced upon the increase of miR-103a-1 levels. Despite these promising results, further studies will have to be performed to demonstrate the mechanistic events linking these miRNAs and the alterations in hERG activity.

## 7. Circulating MiRNAs as Biomarkers for Cardiac Diseases

Due to their presence in various biological fluids, including plasma and serum, and the reliability in their measurements, miRNAs elicited particular attention as biomarkers for many diseases [124]. Several reports have investigated the diagnostic potential of circulating miRNAs as markers for the prediction of cardiovascular morbidity and mortality (Table 1, Figure 3).

Circulating miRNAs were abundantly studied in MI. In a prospective study, 70 patients underwent cardiac magnetic resonance at week 1 and 6 months after ST-segment elevation MI (STEMI) [68]. The analysis of miRNA expression in the plasma at admission revealed the prognostic value of miR-1254, which correlated with left ventricular remodeling and systolic function, such as changes in left ventricular volumes and ejection fraction at 6 months after STEMI. Moreover, circulating miR-22-5p and miR-150-3p were overexpressed in the early stage of acute MI, while miR-132-5p was reduced in plasma of 35 patients with the same condition [69]. Also miR-499 was associated with MI [70] and myocardial damage in cardiac diseases [71], while miR-34a was correlated with left ventricular remodeling after acute MI [72,73]. Finally, circulating miR-30a-5p expression was related with left ventricular dysfunction and heart failure after MI in 99 patients [74].

As far as arrhythmia is concerned, circulating miR-21 was associated with left atrial low-voltages areas in 102 patients with persistent atrial fibrillation undergoing catheter ablation [75], while circulating miR-483, miR-23a and miR-26a may be implicated in post-operative atrial fibrillation [76,77].

Circulating miRNAs have also been linked with inherited cardiomyopathies. MiR-142-5p, miR-143-3p, miR-27b and miR-126-3p were found significantly altered in 30 patients affected with childhood DCM [78]. In a separate study on 55 children affected with DCM, circulating miR-155 and miR-636 were found overexpressed, while miR-646 and miR-639 were downregulated in transplanted patients compared with affected patients who recovered their ventricular function [79]. Also, DCM was associated with altered plasma levels of miR-21, miR-26, miR-29, miR-30, miR-133a [80,81], and with the exosomal miR-92b-5p, found overexpressed in 43 affected patients [82]. Few studies have been performed on ACM, identifying miR-320a and miR-185 significantly down- and upregulated, respectively, in the plasma of affected patients [83,84]. In inherited cardiomyopathies, the circulating miRNA profiling coupled with the harbored mutation can potentially improve the risk stratification of the patients. In the study of Derda et al., increased mir-29a levels were detected in the blood of patients affected with hypertrophic non-obstructive cardiomyopathy carrying mutations in MYH7 gene, while miR-155 was downregulated in HCM patients with MYBPC3 mutations [85]. Also Roncarati et al. associated miR-29a with both hypertrophy and fibrosis in 41 HCM patients, but no correlation with the carried mutation was performed [86].

## 8. MiRNAs as Therapeutic Targets for Cardiac Diseases

The strong impact of miRNAs on the cardiac phenotype elicits particular interest in the possibility of targeting these molecules as therapeutic substrates. Depending on how miRNAs are dysregulated when the heart is upon stress, their individual manipulation is of great potential for novel treatment development to restore the normal phenotype. The technical details of the different therapeutic approaches have been reviewed previously [125]. Here we will summarize the latest data from preclinical studies that have employed viral or polymeric systems.

Recombinant viral systems such as lentiviruses, adenoviruses, and adeno-associated viruses, are commonly used to target genetic material into a given cell. Lentiviral overexpression of miR-99a in a mouse model for MI improved cardiac function via the mTOR/P70/S6K pathway [126]. Also, AAVs have been used to both knockdown or overexpress miRNAs. Tao et al. used AAV9 to transfer miR-433 sponge into the heart of mice after MI, resulting in an improvement of the phenotype [36]. Similarly, miR-378, a regulator of cardiac hypertrophy, was delivered via AAV9 in vivo, improving cardiac function [127]. AAV9 delivery has also been exploited to treat DCM into sarcoglycan-beta (*Sgcb)*-null mouse disease models. The resulting overexpression of miR-669a, downregulated in DCM, reduced cardiac fibrosis, hypertrophy and cardiomyocyte apoptosis for up to 18 months [128].

Different studies focused on the identification of delivery systems based on polymers to modulate miRNAs in specific cardiac conditions. Bejerano et al. delivered intravenously miR-21 mimic to treat MI in mice by using anionic nanoparticles spontaneously assembled upon complexation of the nucleic acid with hyaluronan-sulfate through calcium ion bridges [129]. This strategy succeeded in targeting macrophages in the infarcted area in mouse hearts, leading to pro-inflammatory-to-reparative switch and subsequent angiogenesis, reduced apoptosis, fibrosis and hypertrophy. Another attempt to improve cardiac function after MI includes injectable hyaluronic acid hydrogel miR-302 mimic delivery both in vitro and in a Confetti mouse model [130]. This system promoted cardiomyocytes proliferation and improved cardiac function, such as higher ejection fraction and functional shortening, together with a reduction in cardiac end-diastolic and end-systolic volumes 4 weeks after MI. Hydrogel was used also to deliver polymeric nanoparticles carrying miRNAs (miNPs), such as miR-199a-3p, in human embryonic stem cell-derived cardiomyocytes and endothelial cells or for intramyocardial injection in rats post-MI [131]. As a result, miNPs treatment promoted angiogenesis in hypoxia, with low cytotoxicity in vitro, and improved cardiac function in vivo after 4 weeks. Furthermore, cardiac hypertrophy was improved in mice with aortic coarctation using cholesterol-terminated ethanolamine-aminated poly(glycidyl methacrylate) (CHO-PGEA) to deliver miR-182 inhibitor to cardiomyocytes with a high efficiency [132]. In this context, the delivery of miR-182 inhibitor resulted in the restoration the level of FOXO3, an anti-hypertrophic transcription factor, in the heart of affected animals, which in turn led to alleviation of cardiac hypertrophy, without side effects in other organs.

## 9. Conclusions

Many different biological events interplay to determine the cardiovascular phenotype and its response to injury or stress, with a multitude of miRNAs exerting their influence over these processes. The studies on miRNAs in cardiovascular diseases provide a renewable benefit for the scientific and clinical communities. First, they can help elucidating the pathologic mechanisms by detecting altered target transcripts. The identification of perturbed pathways associated with cardiac phenotypes may allow developing novel drugs to antagonize specific pathogenic mechanisms. Also, in the context of inherited cardiomyopathies, miRNAs could provide insights to study the factors responsible for the wide variable expressivity as well as to bridge the genotype-phenotype gap, thus improving the risk stratification of patients.

Second, several studies identified a number of circulating miRNAs that are altered in the plasma or serum of different subtypes of patients. The potential of these molecules is undisputable to confirm the diagnosis in borderline patients but also to be used as non-invasive early biomarkers and to detect at risk subjects. However, several limitations still hinder their use in the daily clinical practice. Studies on bigger cohorts of patients should be performed to assess the reproducibility of the obtained results. Moreover, standardized protocols should be defined for the detection and the quantification of miRNAs. Finally, cross-comparisons among different patient cohorts should be considered to define the specificity of the miRNA profile for a given disease and so to help in the differential diagnosis. 

Third, miRNAs can potentially be used as a new substrate for novel tailored therapies. Many reports highlight the efficacy of miRNA treatments in vivo; however, further studies are required to translate these results into clinical applications. For example, major effort should be address to test the safety of the delivery systems, the administration, the dosage and the duration of the treatment, as well as the occurrence and the prevention of side effects.

## Figures and Tables

**Table 1:** MicroRNAs involved in cardiac health and disease. Overview of several microRNAs that play a role in different cardiac phenotypes. MI, myocardial infarction; ACM, arrhythmogenic cardiomyopathy; HCM, hypertrophic cardiomyopathy; DCM, dilated cardiomyopathy, LQTS, long QT syndrome.

	**Cardiac miRNA**		
**miRNA**	**Up- or Downregulated**	**Disease**	**Reference**
miR-27a	Upregulated	Contractility	[15]
miR-208a	Upregulated	Contractility	[16,17]
miR-22	Downregulated	Abnormal Ca^2+^ cycling	[18]
miR-1	Upregulated Upregulated Upregulated	Contractility Abnormal Ca^2+^ cycling Chronic heart failure	[19] [20] [21]
miR-133	Upregulated	Chronic heart failure	[21]
miR-17-92 cluster	Upregulated	Arrhythmia	[22]
miR-206	Upregulated	Arrhythmia	[23]
miR-15 family	Downregulated	MI	[24,25]
miR-195	Downregulated	MI	[25]
miR-497	Downregulated	MI	[26]
miR-590-3p miR-199a-3p	Downregulated	MI	[27]
miR-294	Upregulation	MI	[28]
miR-133	Upregulated Downregulated	MI	[29,30] [31]
miR-539	Upregulated	MI	[32,33]
miR-410 miR-495	Upregulated	MI Hypertrophy	[34,35]
miR-433	Upregulated	MI/fibrosis	[36]
miR-22	Downregulated Upregulated	Hypertrophy	[37] [38,39]
miR-212/132 family	Upregulated	Hypertrophy	[40]
miR-199b	Upregulated	Hypertrophy	[41]
miR-199a	Upregulated	Hypertrophy	[42]
miR-206	Upregulated	Hypertrophy	[43]
miR-154	Upregulated	Hypertrophy	[44]
miR-410	Upregulated	Hypertrophy	[35]
miR-495	Upregulated	Hypertrophy	[35,45]
miR-541	Downregulated	Hypertrophy	[46]
miR-1	Downregulated	Hypertrophy	[47,48,49]
miR-133	Downregulated	Hypertrophy	[48,50,51,52]
miR-217-5p miR-708-5p	Upregulated	ACM	[53]
miR-499-5p	Downregulated	ACM	[53]
miR-130a	Upregulated	ACM	[54,55]
miR-21-5p	Upregulated	ACM	[56]
miR-135b	Downregulated	ACM	[56]
miR-204	Upregulated	HCM	[57]
miR-139-5p	Downregulated	HCM	[58,59]
miR-17-5p	Upregulated	HCM	[60]
miR-148a	Downregulated	DCM	[61]
miR-208b	Upregulated	DCM	[62]
miR-451a	Downregulated	DCM	[63]
miR-132	Downregulation	DCM	[64]
miR-155 miR-10b miR-23a	Upregulated	HCM/DCM	[65]
miR-214 miR-21	Downregulated Upregulated	DCM DCM	[65]
miR-1-3p miR-27a	Downregulated	HCM	[65]
miR-1/miR-133a	Downregulated	LQTS	[66]
miR-134 miR-103a-1 miR-143 miR-3619	Upregulated	LQTS	[67]
	**Circulating miRNA**		
**miRNA**	**Up- or Downregulated**	**Disease**	**Reference**
miR-1254	Upregulated	MI	[68]
miR-22-5p miR-150-3p	Upregulated	MI	[69]
miR-132-5p	Downregulated	MI	[69]
miR-499	Upregulated	MI	[70,71]
miR-34a	Upregulated	MI	[72,73]
miR-30a-5p	Upregulated	MI	[74]
miR-21	Upregulated	Arrhythmia	[75]
miR-483	Upregulated	Arrhythmia	[76]
miR-23a miR-26a	Downregulated	Arrhythmia	[77]
miR-142-5p miR-143-3p miR-27b miR-126-3p	Upregulated	DCM	[78]
miR-155 miR-636	Upregulated	DCM	[79]
miR-646 miR-639	Downregulated	DCM	[79]
miR-29	Upregulated	DCM	[80,81]
miR-21 miR-26 miR-30 miR-133a	Downregulated	DCM	[80,81]
miR-92b-5p	Upregulated	DCM	[82]
miR-320a	Downregulated	ACM	[83]
miR-185	Upregulated	ACM	[84]
miR-29a	Upregulated	HCM	[85,86]
miR-155	Downregulated	HCM	[85]
